# Establishing a Specialty Bipolar Clinic: The National Network of Depression Centers Consensus Recommendations

**DOI:** 10.1111/bdi.70154

**Published:** 2026-07-10

**Authors:** Christopher D. Schneck, Jorge R. C. Almeida, David J. Bond, Katherine E. Burdick, Mark A. Frye, Fernando S. Goes, Rebekah S. Huber, Rodrigo Machado‐Vieira, Pamela Mahon, Melvin G. McInnis, John Nurnberger, Dost Ongur, Michael J. Ostacher, Sagar V. Parikh, Kristin S. Raj, Matteo Respino, Barbara W. Schweizer, Jair C. Soares, Sarah H. Sperry, Aimee E. Sullivan, Trisha Suppes, Marin Veldic, Peter P. Zandi, J. Raymond DePaulo

**Affiliations:** ^1^ Department of Psychiatry University of Colorado Anschutz Aurora Colorado USA; ^2^ Department of Psychiatry and Behavioral Sciences Dell Medical School, the University of Texas at Austin Austin Texas USA; ^3^ Department of Psychiatry Johns Hopkins School of Medicine Baltimore Maryland USA; ^4^ Department of Psychiatry Columbia University New York New York USA; ^5^ Department of Psychiatry Mayo Clinic College of Medicine Rochester Minnesota USA; ^6^ Department of Psychiatry, Department of Psychiatry Steven J. Sharp Center for Mental Health Innovation, Oregon Health and Science University Portland Oregon USA; ^7^ Department of Psychiatry University of Texas Houston Houston Texas USA; ^8^ Department of Psychiatry Mass General Brigham Boston Massachusetts USA; ^9^ Department of Psychiatry University of Michigan Ann Arbor Michigan USA; ^10^ Department of Psychiatry Indiana University School of Medicine Indianapolis Indiana USA; ^11^ Department of Psychiatry Stanford University School of Medicine Stanford California USA; ^12^ Department of Psychiatry University of British Columbia Vancouver British Columbia Canada

**Keywords:** bipolar disorder, collaborative care, guideline‐concordant care, measurement based care, psychoeducation, psychosocial interventions, treatment adherence

## Abstract

**Objective:**

To provide expert recommendations for the development of a specialty bipolar clinic that promotes best clinical practices for the delivery of safe and effective care.

**Methods:**

Members of the National Network of Depression Centers (NNDC) Bipolar Clinics Work Group met in a series of 1‐h monthly video calls over 18 months to discuss all aspects of their respective institution's bipolar clinics, including services provided, administrative processes, staffing models, characteristics of patients treated, patient volumes, finances, and research. The group also invited psychiatrists from outside of the NNDC who ran large bipolar clinics to contribute their thoughts and experiences on the subject. In addition, the NNDC sent a 20‐question survey to all 27 sites asking clinic directors specific questions about their bipolar clinics, if applicable. Further meetings and discussions incorporating results from the survey took place to produce a comprehensive set of recommendations.

**Results:**

Twenty‐one of 27 NNDC sites (81%) responded to the survey. Twelve of 21 (57%) sites had a specific bipolar clinic, and another 4 (19%) were looking to start one. Clinics were based in university settings. The meetings, clinic survey, and discussions resulted in 10 recommendations describing services and operations for bipolar specialty clinics.

**Conclusions:**

Given the complexity of bipolar disorder, specialty clinics are important to enhance care. Clinics with a wide array of services that include consultation, medication management, bipolar‐specific psychotherapies, and patient/family psychoeducation provide the most comprehensive care. Challenges to these clinics include maintaining ongoing patient access as well as ensuring long‐term financial stability.

## 1 Introduction

Bipolar disorder is a complex and chronic mental health condition characterized by extreme mood swings, including episodes of mania and depression. Individuals with bipolar disorder commonly suffer from comorbid psychiatric conditions, most often anxiety disorders, substance use disorders, personality disorders, and attention deficit hyperactivity disorders [1]. In addition, bipolar disorder is associated with a high prevalence of medical conditions, including obesity, diabetes, hypertension, cardiovascular disease, and migraines [2]. The combination of the chronic nature of the disease and the frequent psychiatric and medical comorbidities all contribute to the high morbidity and premature mortality of the illness [2].

There is evidence that treatment in a specialized outpatient bipolar disorder clinic, especially early in the course of the illness, substantially reduces readmission rates to psychiatric hospitals and increases satisfaction in care [3]. Optimal treatment of bipolar disorder involves measurement‐based assessments, guideline‐concordant medication management and the use of evidence‐based psychosocial treatments specific for bipolar disorder [1]. Patients with bipolar disorder often require a higher frequency of appointments than those suffering from other psychiatric disorders (e.g., unipolar depression) and may require urgent appointments depending on the instability of their illness, especially if heading into mania. Due to the relapsing and remitting nature of the illness, a long‐term, multidisciplinary approach is most often needed to ensure optimal outcomes [1].

The National Network of Depression Centers (NNDC), a consortium of 27 geographically distributed academic medical centers providing care for patients with mood disorders, formed the NNDC Bipolar Disorders Clinics Work Group 2 years ago. The goals of this Work Group were several‐fold: to understand how bipolar clinics functioned within and outside of the network, to share strategies and methods to improve care, and to decide on best clinical practices for the delivery of safe and effective care in this population. The group also invited psychiatrists from outside of the NNDC who ran large bipolar clinics to contribute their thoughts and experiences on the subject. This article reports on the work of these efforts in promoting best clinical practices for a bipolar clinic in order to optimize the delivery of safe and effective care.

## 2 Methods

As part of creating recommendations about best practices in a bipolar clinic, members of the Bipolar Clinics Work Group participated in a series of 1‐h monthly video conference calls over 18 months, discussing their respective institution's bipolar clinic, its operations, services provided, administrative processes, staffing models, characteristics of patients treated, patient volumes, finances, and research (if applicable). The group also discussed challenges their clinics faced (e.g., staffing, access, patient capacity, finances, training, etc.), lessons they had learned to optimize care or flow of their patients, and what changes they would like to make to improve their clinics in the future.

In addition, the group conducted a survey across all 27 of the NNDC network sites. The 20‐question survey first asked clinical directors if their institution had a specific clinic for bipolar disorder and, if so, detailed questions about the clinic's structure, patients, providers, trainees, models of care, types of care provided, ability to see new patients, length of treatment, payer sources, research, and financial structure. We also held discussions with present and past clinic directors from outside of the NNDC, including UCLA and Massachusetts General Hospital (now Mass General Brigham) to gather their perspectives on the same set of subjects.

The AI OpenEvidence was used for the purposes of literature searches.

## 3 Results

Twenty‐one of 27 NNDC sites (81%) responded to the survey. Centers that responded included the following: Brigham and Women's, Duke University, Dell‐UT Austin, Emory University, University of Colorado Anschutz Medical Campus, Indiana University School of Medicine, Johns Hopkins University, the Mayo Clinic, McLean Hospital, Michigan State University, Ohio State University, Penn State University, Stanford University, University of Cincinnati, University of Florida, University of Illinois Chicago, University of Iowa, University of Michigan, UT Houston, Weill Cornell, and University of Utah.

Twelve of 21 (57%) of the sites had a specific bipolar clinic, and another 4 (19%) were looking to start one in the near future. The number of patients treated in each clinic ranged from 90 up to 800, with a mean of 239 patients. All 12 clinics surveyed accepted Medicare and private insurance; 10 accepted Medicaid; 8 accepted other Federal insurance (e.g., Tricare); and 2 clinics accepted self‐pay as a payment option.

Incorporating our monthly discussions and responses from the survey, what follows are the characteristics an ideal bipolar clinic would possess, as well as common challenges these clinics face and further ideas as to how clinics can be enhanced in the future (see Table 1).

**TABLE 1:** Summary of consensus recommendations.

Topic	Comments
1. Bipolar clinics should use standardized measures for assessing patients	Incorporating measurement‐based care improves outcomes of patients with bipolar disorder Using scales to systematically assess symptoms is highly acceptable to patients and providers Self‐rated scales and those in the public domain are generally preferable for ease‐of‐use and cost‐savings Mood charting is an important component of measurement‐based care in bipolar disorder
2. Screening patients referred to a bipolar clinic is preferred	Given the frequency of misdiagnosis of bipolar disorder, screening tools (e.g., the MDQ) or use of a triage clinician should be considered. Clinics will need to determine the relative cost/benefit they receive by screening all patients. Creating processes to transfer patients out of clinic who are not a good diagnostic fit should be considered a priori to aid in patient through‐put
3. Clinics should treat the entire diagnostic spectrum of bipolar disorder, including early intervention strategies for those at high risk for bipolar disorder	Given the heterogeneity in presentations and course of illness, clinics should treat the full spectrum of Bipolar Disorders (bipolar I, bipolar II, specified/unspecified bipolar disorders) Broadening the age range from pediatric to adolescents to adults maintains continuity of care but comes at additional costs to add specialty providers (i.e., child trained)
4. Consultation, medication management, bipolar‐specific psychotherapy and group psychotherapy should be offered, including group medical visits	One‐time consultation services can extend the reach of bipolar expertise into communities that might not otherwise be able to access such care Ongoing medication management is a core component of treatment in bipolar disorder, as are bipolar‐specific psychotherapies such as IPSRT, FFT and CBT Group medical visits provide another way to expand access to bipolar clinics, can enhance resident education and provide flexibility for urgent visits.
5. Education to patients, families and the public is an important component of overall psychoeducation of bipolar disorder	Psychoeducation is a core component of all bipolar psychotherapies and can benefit patients and families to improve outcomes Multiple modalities can be effective, whether individual therapy, group therapy, peer support, workbooks or digital supplements (programs, apps) Improving knowledge about bipolar disorder can reduce stigma
6. Maintaining throughput of patients is important for ongoing access to specialty care, though transferring patients out of clinic can be difficult	Transferring patients out of a bipolar clinic is often contingent upon multiple factors: patient stability, insurance, community provider availability, primary care providers' willingness to accept managing potentially complex psychiatric patients even when stabilized Managing patient expectations upon clinic entry can aid in transferring patients out of clinic (i.e., noting at the outset that care is time‐limited)
7. A multidisciplinary staffing model allows for a greater array of services	Use of psychiatrists, advanced practice providers, psychologists, social workers, pharmacists and other behavioral health providers can allow for better individualized treatment, shared decision‐making and better integration of medical, psychological and social support
8. The inclusion of trainees from many disciplines can expand access to care and foster the next generation of clinicians skilled in managing complex mood disorder patients	Under supervision, trainees can participate in medication management, psychotherapy, symptom monitoring, care coordination, etc. and act as “provider multipliers” to extend care Insurance can be a potential barrier as to whether or not they will pay for supervised trainees to provide care
9. Having multiple funding streams improves the long‐term viability of the clinic, at least in academic settings	Relying solely on clinical revenue to support operations is difficult. Diverse revenue sources such as from grants, hospital/institutional support, grant‐funding and research can improve long‐term sustainability
10. Having a “clinical champion” dedicated to seeing the clinic progress, as well as having administrative buy‐in and support are essential for clinic viability	Successful development of a bipolar clinic depends on a clearly designated clinical leader capable of navigating administrative, clinical, and educational challenges to maintain momentum Deciding on needed services a priori and then developing staffing models and administrative support are needed, depending on the likely number of patients who will access the clinic

Abbreviations: CBT, Cognitive Behavioral Therapy; FFT, Family Focused Therapy; IPSRT, Interpersonal and Social Rhythm Therapy; MDQ, Mood Disorder Questionnaire.

### 3.1 Bipolar Clinics Should Use Standardized Measures for Assessing Patients

All clinics that responded (8 of 12, or 67%) use some type of measurement‐based care in their evaluation of patients. The range of scales used across various institutions was notable: across the 12 sites, a total of 15 different scales were administered to patients. The PHQ‐9 and GAD‐7 were used by the majority of bipolar clinics (8 of 10, or 80%) to assess depression and anxiety, respectively. While clinicians acknowledged the two scales' shortcomings (e.g., they were not created specifically for bipolar disorder and each measures the frequency of symptoms but not intensity), all also agreed that accessibility (the scales were widely available at their respective institutions and incorporated into their electronic medical records) and lack of cost (they are in the public domain) were primary determinants in their use. All clinicians agreed that assessment of mania, while important, was far more difficult to assess in a non‐research setting, given the need to use a self‐report measure and not a clinician‐administered one. Only two sites reported using the Altman Self Rated Mania Scale, and all clinicians felt that the scale was poor in its usefulness as a clinical decision‐making tool. One site reported using the Patient Mania Questionnaire‐9 (PMQ‐9), noting it was helpful in picking up subthreshold or hypomanic symptoms, but less useful when manic symptoms were more severe. Clinicians also agreed that incorporating a functional measure was important to better assess a patient's overall well‐being, though only 2 sites used them (the Work and Social Adjustment Scale (WSAS) and the World Health Organization Disability Assessment Schedule (WHODAS)).

Numerous studies have shown superior outcomes when incorporating measurement‐based care in the management of patients with psychiatric illness [4]. Using scales to systematically assess symptoms is highly acceptable to patients and providers, helps inform and drive clinical decision‐making, and can be used for quality improvement projects. There are presently 28 scales specifically designed to assess symptoms in bipolar disorder: 10 scales assess manic symptoms, 8 assess depressive symptoms, and 10 assess both manic and depressive symptoms [5]. While measures such as the Altman Self‐Rating Mania Scale, the Bech‐Rafaelsen Mania Rating Scale, the Quick Inventory of Depressive Symptomatology, the Internal State Scale, and the Bipolar Inventory of Symptoms Scale may have high clinical utility [5], the choice of measures in clinical settings will likely be driven primarily by cost, availability, ease of use, and brevity. Use of functional measures is clearly important but under‐used: in a survey of members of the Depression and Bipolar Support Alliance (DBSA), nearly 90% of patients felt that measures of quality of life and personal functioning were most important to track over time [6, 7]. Mood charting, either using digital apps or paper‐and‐pencil methods, can help improve outcomes by enhancing patient self‐awareness, aiding in early detection of mood changes, and facilitating better communication with providers [8, 9].

### 3.2 Screening Patients Into a Bipolar Clinic is Preferred, Though Can Be Difficult to Implement and Maintain

The majority of bipolar clinics (7 of 12, or 57%) openly accepted referrals to their clinic for any patients who had been diagnosed with bipolar disorder or were suspected of having bipolar disorder, without further screening for accuracy of diagnosis. Other clinics used a variety of screening methods prior to establishing an intake visit. Several clinics used questions on an intake form regarding past diagnosis, past treatment, and hospitalizations to inform proper placement into their clinics. Other sites commented that many of their referrals came from their own inpatient units and therefore felt that the diagnosis of bipolar disorder carried a high degree of reliability. Three of the clinics (25%) used a clinician to screen new referrals, and two clinics used an assessment tool or scale. One site noted it had previously tried to screen patients for proper or likely diagnosis using a clinician, but the sheer volume of screening and the frequency in which the diagnosis appeared likely or highly suspicious led them to abandon their screening procedures and openly take all referrals.

Given the frequency of misdiagnosis of bipolar disorder and/or the delay in reaching an accurate diagnosis, screening for entry into a bipolar clinic is an attractive option [1, 10]. Screening tools such as the Mood Disorder Questionnaire or the Bipolar Spectrum Diagnostic Scale may be useful in helping to establish a bipolar diagnosis, though the specificity and sensitivity of these tools can vary depending on the population in which they are used [11, 12]. Decisions on whether or not to screen patients for intake into a bipolar clinic and how best to screen will likely be determined by the cost and the relative value a clinic feels they are getting from screening all referrals into their clinics. Successful screening is more likely to be accomplished by an individual with clinical training (e.g., social worker, nurse, licensed professional counselor, etc.) but the cost of staffing such a position for unreimbursed time versus overall benefit to the clinic will need to be considered on a clinic‐by‐clinic basis. Lastly, having a clear process for transferring patients to a different clinic if they are found not to have bipolar disorder is also necessary and should be considered a priori, as well as having clear processes for internally transferring patients from general psychiatric clinics into ones that provide bipolar‐specific care.

### 3.3 Clinics Should Treat the Entire Diagnostic Spectrum of Bipolar Disorders Including Early Intervention Strategies for Those at High Risk for Bipolar Disorder, if Feasible

The majority of clinics (10 of 12 or 83%) treated the full spectrum of Bipolar Disorders (BD I, BD II and unspecified). Two clinics treated only patients with bipolar I disorder, primarily due to research demands with the need to recruit a specific bipolar population. Nine of the 12 clinics (75%) provided treatment for ages 18 and up, 1 clinic provided care for teens 13–17 as well as adults, and 1 clinic provided care for pediatric, teen and adult bipolar patients. No clinic imposed an upper age limit for care, that is, there was no separate geriatric psychiatric clinic. One clinic offered an additional early intervention program for youth who were showing symptoms of a mood disorder and/or were at high risk for bipolar disorder by virtue of having a first‐degree relative with bipolar disorder, with the patient developing mood symptoms.

Given the heterogeneity in presentations and course of bipolar disorder, treating the full spectrum of the illness makes sense for clinics unless the clinic is primarily research‐driven. Broadening the age range to include pediatric and adolescent patients helps improve access to care and maintains continuity of care, though it adds clinical, administrative and hiring complexities by requiring providers with specific training in managing mood disorders in youth. However, treating a wider range of bipolar illnesses, that is, more bipolar spectrum illnesses, may increase the potential for misdiagnosis, and lead to an increased need to transfer patients out of the clinic. It may also lead to the increased prevalence of trauma‐related and/or personality disorders within the clinic [2].

The high heritability of bipolar disorder, the fact that the majority of adults report the onset of their illness prior to age 18 [13], and the concept that bipolar disorder is a neuroprogressive disorder all support the clinical utility in creating a high‐risk or early intervention clinic for bipolar disorder [14]. Earlier illness onset and longer delays prior to first treatment are associated with longer and more severe depressive phases, more comorbid disorders and less time well in adolescence or early adulthood [15]. There is increasing evidence that early intervention in the illness can improve outcomes over time and lead to changes in brain connectivity that improve emotion regulation [16]. While novel, such clinics provide an important opportunity to potentially change the course of the illness [17] and lower its morbidity and mortality.

### 3.4 Consultation, Ongoing Medication Management, Bipolar‐Specific Psychotherapy and Group Psychotherapy Should Be Offered. Group Medical Visits Should Strongly Be Considered

Offering a full array of clinical services for bipolar patients is ideal though it can be difficult to implement and sustain. All clinics (12 out of 12) offered medication management in the treatment of patients with bipolar disorder, and 11 of 12 (92%) offered one‐time consultations. Seven of 12 offered bipolar‐specific therapy for bipolar disorder, and one clinic offered several types of non‐specific therapies for bipolar disorder. Five of 12 (42%) offered group psychotherapy, most in addition to individual therapy, and these ranged from supportive group therapy, educational groups about bipolar disorder, or more general psychotherapy groups. One clinic offered group therapy via peer specialists. Three clinics (25%) offered group medical visits, also known as shared medical appointments, in an effort to expand their ability to see larger numbers of medication management patients and offer a unique training experience to residents (see below) [18].

Medication management is central to the treatment of patients with bipolar disorder, often encompassing both acute/urgent stabilization and long‐term maintenance. Pharmacotherapy—primarily with mood stabilizers such as lithium and lamotrigine, as well as antipsychotics and adjunctive agents—remains the foundation of evidence‐based care, with lithium considered the gold standard for both bipolar I and II disorders due to its efficacy in preventing manic and depressive recurrences, as well as its anti‐suicidal properties [1]. Ongoing monitoring is essential to assess efficacy, detect side effects (such as lithium‐induced renal or thyroid dysfunction), and address adherence. Nonadherence rates are high, often due to adverse effects, regimen complexity, lack of insight, and/or stigma. Improvements in adherence may be helped by simplifying regimens, ongoing psychoeducation and open discussions regarding patient beliefs and attitudes regarding medications, life circumstances and perceived benefits and disadvantages of pharmacotherapy [19].

Performing one‐time consultations can be an important strategy in extending specific bipolar expertise to a larger number of patients who otherwise might not be able to access a bipolar clinic's services. One‐time consultations provide an opportunity for expert diagnostic clarification, comprehensive assessment of mood symptoms, comorbidities, and broader differential diagnoses, including medical and psychiatric conditions that may mimic bipolar disorder [1]. Additionally, one‐time consultations can deliver targeted psychoeducation to patients and families, improving understanding of the illness, the importance of medication adherence, and early recognition of relapse, all of which are associated with reduced relapse rates and improved long‐term outcomes [1]. For clinics that use a team‐based care approach and clinical conference discussions, one‐time consults can further benefit patients by incorporating opinions from varied disciplines (psychiatrists, psychologists, pharmacists, etc.). Finally, consultations can be especially important when patients are living in rural communities in which access to mood disorder specialists is limited or non‐existent [20]. Such consultations may then provide guidance on pharmacotherapy and/or psychosocial interventions that can help primary care providers or referring clinicians implement evidence‐based management strategies.

Bipolar‐specific psychosocial treatments combined with pharmacotherapy can improve patient outcomes and give greater treatment options for patients beyond pharmacotherapy. Adjunctive psychoeducation, cognitive behavioral therapy (CBT), family‐focused therapy (FFT), or interpersonal and social rhythm therapy (IPSRT) should be considered for acute depression and maintenance treatments, as well as peer support for maintenance [21, 22]. There is some evidence that individuals with bipolar disorder do better when they receive treatment they prefer, inclusive of psychotherapy [21], and patients have indicated that alternatives and adjuncts to pharmacotherapy are a highly desired aspect of treatment [23]. That being said, clinics may be financially challenged to incorporate psychotherapy while maintaining a model that is financially sustainable. Online psychotherapy in various forms (e.g., cognitive behavioral therapy for bipolar depression, psychoeducation, structured self‐management programs) has shown efficacy as an adjunctive component in the treatment of bipolar disorder, especially when integrated with multidisciplinary care and medication management in a bipolar clinic [24, 25].

Group Medical Visits (GMVs), also known as Shared Medical Appointments, potentially provide an excellent way to expand access to a bipolar clinic, enhance resident education and provide flexibility for urgent visits [18]. As developed at the University of Michigan, patients are seen in a group format run by a staff psychiatrist and a resident in psychiatry, where medication adjustments can take place while providing an educational opportunity for patients about treatments, side effects and options. A survey of patients participating in GMVs has shown broad patient satisfaction, and residents have noted GMVs to be “definitely helpful” in interviewing skills, clarifying diagnosis and learning treatment approaches. Challenges to this approach are commonly patients' discomfort with the group format and patient concerns about privacy. However, given the high rates of patient satisfaction and access issues most bipolar clinics face, GMVs should be considered as an adjunct to care offered in bipolar clinics.

### 3.5 Education to Patients, Families and the Public is an Important Component of Overall Psychoeducation of BD

The majority of NNDC bipolar clinics (7 of 12, or 58%) offered some type of education about the illness to patients, families, or the community on bipolar disorder. The range of such offerings included a dedicated educational program for patients, families, and providers (including community lectures, a website, provider training, and other educational resources) to psychoeducation visits with a nurse, a monthly family support group run by a social worker, talks in the community, social media campaigns, general psychoeducational groups, and the Life Goals Psychoeducational Group based on the Bauer and McBride Manual [26].

Psychoeducation is a core component of nearly all bipolar‐specific therapies, teaching skill development, early detection in managing prodromes of depression and mania, ongoing stress management, problem‐solving, and dealing with the effects of stigma and denial of the illness [1]. Multiple modalities can be effective, including individual therapies, peer support, group psychoeducation, and newer models involving online tools, smartphone apps and workbooks [27]. The array of effective delivery methods should allow clinics to find some modality of psychoeducation that can work within a particular clinic's patient, administrative and financial structures, especially as internet, app‐based and computer‐based programs are becoming more widespread. Internet‐based and app‐based programs that provide structured psychoeducation, self‐management tools, mood and medication tracking have shown evidence in improved outcome and patient acceptability and extend access to important components of treatment [25, 28, 29, 30]. However, our experience has been that such online programs (e.g., MoodSwings [28], ORBIT [29], SIMPLe [30] and LiveWell [25]), while shown to be effective, often have a time‐limited existence, such that referring to a validated, reliable and ongoing program may be challenging.

### 3.6 Maintaining Through‐Put of Patients is Important, Though Transferring Patients Out of Clinic Is Difficult

Bipolar clinics operated at capacity, and 7 of 12 (58%) clinics reported having a waitlist to gain admission into their clinics. The waiting time to access services ranged from 1 month to 4 months, with several clinics reporting a two‐month waiting time. Despite access issues, only 4 of 12 clinics (33%) set time‐limits to the length of treatment a patient could receive, though even these clinics acknowledged there were always patients who required ongoing specialty care and remained in the clinic on a longer‐term basis. One site had established a “First Episode” clinic (i.e., patients who had experienced their first manic episode) with their treatment program lasting 2–3 years. Patients were often then transferred to a “chronic patient clinic” that had no limits to the length of treatment. Another clinic noted it “treats toward stability” with treatment generally lasting 12–14 months. Patients would then be transferred either out to the community or back to their PCPs, though some remained in clinic due to their chronically unstable disease. Clinics with a shorter treatment model noted the importance in explaining the time‐limited nature of their treatment at the outset in order to level‐set patient expectations.

Transferring patients out of the clinic was noted to be difficult for a variety of reasons. Patients themselves were very reluctant to leave, as many clinics noted that patients experienced a sense of relief being seen in a specialty clinic in which providers and staff had a deep understanding of their illness, coupled with the provision of bipolar‐specific therapies. Transferring patients into community settings or to primary care physicians was a common strategy, though frequently difficult to do. Nine of 12 clinics (75%) were able to transfer patients into the community for ongoing care, more often to primary care physicians within their networks. Success of transfers was largely contingent upon the stability of the patient and the simplicity of the medication regimen, and several clinics noted that PCPs were often reluctant to take patients on lithium. More successful transfers to PCPs were linked to the promise of taking back the patient should the patient become destabilized. Lastly, insurance coverage in clinics was broadly available, but insurance barriers were common outside of clinic settings, as many community psychiatrists did not accept private insurance, Medicaid or Medicare, and patients were unable to afford fee‐for‐service care.

Access to care remains a chronic challenge for psychiatric patients in general and patients with bipolar disorder in particular [31]. Surveys of patients with BD have found access to quality and specific care an area of primary concern, especially for those who are economically disadvantaged and/or living in rural areas [23]. Time‐limited treatment models in bipolar clinics allow for greater access to expert care, but at the potential expense of higher relapse rates and reduced patient satisfaction [3].

### 3.7 Having a Multidisciplinary Clinical Staff Allows Provision of Greater Types of Services

All bipolar clinics had multidisciplinary providers, though clinics varied widely in the types of disciplines on staff. All clinics had psychiatrists, 8 of 12 (67%) had psychologists, 7 of 12 (58%) had advanced practice providers (NPs or PAs), 4 of 12 had social workers (25%), and 2 of 12 (17%) had pharmacists. Only one clinic was staffed by psychiatrists alone.

A multidisciplinary team‐approach offers significant advantages by providing comprehensive coordinated care that addresses the complex needs of individuals with bipolar disorder. Having psychiatrists, psychologists, nurse practitioners, social workers, pharmacists, and other behavioral health providers working together allows for better integration of medical, psychological, and social support. A team‐based model facilitates more accurate diagnosis, individualized treatment planning, and more consistent monitoring of medication and therapy effectiveness. Such collaboration can support shared decision‐making and enhance patient engagement by providing a wide array of care: medication management, therapy, crisis intervention, and support with housing or employment. Use of advanced practice providers can potentially improve access to care by providing medication management and psychoeducation at lower costs than psychiatrists. Depending on how interdisciplinary team meetings are run, some clinics will review a list of patients seen in the last week or month and highlight particularly complex patients that can benefit from further discussion. Research has demonstrated that using multidisciplinary teams improves clinical outcomes, better functional recovery, and higher patient satisfaction compared to usual care [3, 32, 33, 34].

### 3.8 The Inclusion of Trainees Such as Psychiatric Residents, Medical Students, Nursing Students, Psychology Interns and Externs, Social Work Interns and Externs Can Expand Access to Patient Care and Foster the Next Generation of Clinicians Skilled in Managing Complex Mood Disorders

Given that all clinics surveyed were located at academic medical centers, unsurprisingly, all clinics (12 of 12) had trainees in their clinics. All clinics had psychiatric residents and nearly all (10 of 12, or 83%) had medical students. Four of the clinics (33%) had MD fellows, four had psychology interns or externs, two had social work interns or externs, and two had nurse practitioner students.

Having trainees of multiple disciplines in bipolar clinics can increase access to psychiatric care while educating and expanding the future workforce. Under supervision, non‐prescriber trainees can participate in psychotherapy/psychoeducation, symptom monitoring, and care coordination, all of which are core components of collaborative care models shown to improve outcomes in bipolar disorder [33]. Residents, medical students, and advanced practice provider students increase their ability to diagnose and care for complex mood disorder patients. Trainees from diverse disciplines can deliver or support evidence‐based psychotherapies under supervision—including CBT, family‐focused therapy, IPSRT, and mindfulness‐based interventions—thereby expanding the clinic's ability to offer these treatments to a larger patient population and reducing wait times. Therapy trainees (psychology interns/externs, social work interns/externs/fellows) can often see patients at reduced costs, and many private insurances allow for trainees to provide care (however, Medicare typically does not, and Medicaid allowances are usually determined state‐by‐state).

### 3.9 Having Multiple Funding Streams (e.g., Clinical Revenue, Institutional Support, Grants/Research, and Philanthropy) Improves the Long‐Term Viability of the Clinic, at Least in Academic Settings

Clinics surveyed reported multiple funding streams to support their finances. Eight of 12 clinics (67%) conducted research that was either grant‐funded or funded by means other than grants. Three of 12 clinics (25%) conducted industry‐sponsored trials, and 8 received other grant or philanthropic support apart from research funding. Four of the clinics (33%) received hospital support to add to their financial viability. While most clinics reported that they were financially sustainable, several commented that good philanthropic support was essential to their financial stability, and few felt they could sustain themselves solely from clinical revenues.

Key considerations in the financial modeling of outpatient psychiatric clinics providing multidisciplinary care for bipolar disorder include staffing mix, supervision and trainee infrastructure, reimbursement models, and patient volume. As all of the clinics surveyed were at academic centers that typically have higher overhead expenditures (e.g., staffing models that require supervision of trainees and clinic models that require structured training programs), the need for outside revenue sources and support is not necessarily surprising. Academic centers have greater access to institutional support, grant funding, and value‐based payment arrangements which can potentially offset these expenditures, while private clinics are more dependent on fee‐for‐service billing and may face greater challenges with insurance reimbursement for multidisciplinary services. Private outpatient clinics often use leaner staffing models and have no larger institutional costs to support, typically resulting in lower fixed costs, though this may limit the range and intensity of services offered, particularly for evidence‐based psychotherapies that require specialized training. Long‐term financial sustainability in both settings depends on efficient schedule management, patient through‐put, reimbursement rates from third‐party payers, and balancing delivery of specialty care vs. costs of such programs that increase overhead [35].

### 3.10 Having a “Clinical Champion” Who is Dedicated to Seeing the Clinic Move Forward, as Well as Having Administrative Buy‐In and Support Are Essential for Clinic Viability

All sites with bipolar specialty clinics agreed that having a clear clinical lead to keep the clinic on‐track, as well as having solid administrative support were essential in starting and maintaining a bipolar clinic. Group members who had developed bipolar clinics at their institutions recognized the importance of having a clearly designated faculty leader who could problem‐solve efficiently, collaborate across administrative, clinical, and educational domains, and sustain focus on the clinic's development. Without such a clinical lead, members agreed that the inherent complexities and institutional and insurance changes often jeopardize the clinic's formation or its long‐term sustainability.

Other important pre‐launch steps for the creation of a bipolar specialty clinic include knowing the number of patients who are likely to benefit from the service, deciding on the frequency of clinic days, the types of services the clinic wants to offer (e.g., medication management, consultations, psychotherapy, etc.) and then deciding on the staffing model to support the services. Given the nature of the illness, working to ensure rapid and flexible access to care can potentially reduce emergency room visits, hospitalizations and improve treatment adherence [36]. Having a care coordinator who facilitates triaging and screening of referrals, pre‐visit paperwork, and, if possible, direct scheduling of intakes can improve workflow processes, though the benefit of the position must be balanced by staffing a role with no direct care reimbursement. In addition, ready access to higher levels of care, such as intensive outpatient programs (IOPs) and partial hospital programs (PHPs) can help bridge the gap between inpatient hospitalization and standard outpatient management. For those clinics that do not have internal system IOPs and PHPs, building relationships with external programs can facilitate rapid referral of bipolar patients to those programs and likely decrease hospitalization rates [37, 38].

## 4 Conclusion

Given the complexity of bipolar disorder, its chronic course, and the frequency of comorbid psychiatric and medical conditions, bipolar specialty clinics are important to enhance care and have been shown to improve patient outcomes. Clinics with a wide array of treatment services that include consultation, medication management, bipolar‐specific psychotherapies, and patient/family psychoeducation offer patients the most comprehensive care. Ideally, bipolar clinics treat the full spectrum of bipolar disorder, including early onset disorders and patients at high risk for bipolar disorder. Multidisciplinary teams, often composed of psychiatrists, advanced practice providers, psychologists, and social workers, offer significant advantages by providing comprehensive coordinated care that includes shared decision‐making, enhanced patient engagement, and management of both acute and long‐term patient needs. Including trainees of multiple disciplines can increase access to psychiatric care while educating and expanding the future workforce. Challenges to these clinics include maintaining throughput of patients to optimize access as well as ensuring ongoing financial stability. Having solid administrative support is critical in ensuring the success of a bipolar specialty clinic.

Limitations of this review include those inherent to the endeavor of bringing a finite group of experts together in an attempt to consolidate a large amount of data and experiences into a single document. All experts in the group were from academic university settings in North America and therefore generalizability into other non‐academic and non‐North American geographic settings may be limited. However, most of the recommendations were not felt to be limited to academic settings, such as using measurement‐based care, treating the full spectrum of bipolar illness, providing bipolar‐specific psychotherapies and medication management/consultation services, maintaining throughput of patients, creating multi‐disciplinary teams and having solid administrative support. Recommendations about use of trainees and financial support from hospitals/research grants are much more specific to academic institutions and are therefore less generalizable, especially in community settings. Our recommendations serve primarily as suggestions for good clinical practice based on expert opinion and consensus of the authors at the time of this writing.

## Data Availability

The data that support the findings of this study are available from the corresponding author upon reasonable request.
